# Antibody Phage Display Technology for Sensor-Based Virus Detection: Current Status and Future Prospects

**DOI:** 10.3390/bios13060640

**Published:** 2023-06-09

**Authors:** Olga I. Guliy, Stella S. Evstigneeva, Vitaly A. Khanadeev, Lev A. Dykman

**Affiliations:** Institute of Biochemistry and Physiology of Plants and Microorganisms, Subdivision of the Federal State Budgetary Research Institution Saratov Federal Scientific Centre of the Russian Academy of Sciences (IBPPM RAS), 13 Prospect Entuziastov, Saratov 410049, Russia; guliy_olga@mail.ru (O.I.G.); stels20295@yandex.ru (S.S.E.); dykman_l@ibppm.ru (L.A.D.)

**Keywords:** biosensors, viruses, detection methods, antibody phage display technology

## Abstract

Viruses are widespread in the environment, and many of them are major pathogens of serious plant, animal, and human diseases. The risk of pathogenicity, together with the capacity for constant mutation, emphasizes the need for measures to rapidly detect viruses. The need for highly sensitive bioanalytical methods to diagnose and monitor socially significant viral diseases has increased in the past few years. This is due, on the one hand, to the increased incidence of viral diseases in general (including the unprecedented spread of a new coronavirus infection, SARS-CoV-2), and, on the other hand, to the need to overcome the limitations of modern biomedical diagnostic methods. Phage display technology antibodies as nano-bio-engineered macromolecules can be used for sensor-based virus detection. This review analyzes the commonly used virus detection methods and approaches and shows the prospects for the use of antibodies prepared by phage display technology as sensing elements for sensor-based virus detection.

## 1. Introduction

The concept of a virus often raises public concern about the spread of the new coronavirus infection, dengue fever, avian influenza virus, hepatitis, Ebola fever, acquired immunodeficiency syndrome (AIDS), and other deadly diseases [1,2]. However, despite their enormous harmfulness, many viral and virus-like particles have practical applications in bio(nano)medical technologies. In particular, they can be used in targeted drug delivery, in the transfer of genetic material [3,4], in noninvasive imaging for the early detection and treatment of human diseases [5], as biorecognition elements in biosensor systems [6,7], and in the production of vaccines and antimicrobials [8]. Among other things, viral particles can be used in bioelectronics to form certain functional surfaces and materials [4,9], owing to their ability to genetically adapt to any changes from the outside. For instance, icosahedral viral nanoparticles such as tobacco mosaic virus and bacteriophage M13 have been used as nanotubes for batteries and nanowires, while rod-shaped viral nanoparticles such as cowpea mosaic virus and cowpea chlorotic mottle virus have been considered for use in biomedicine [5,10,11]. Additionally, it has been confirmed that archaeal *Sulfolobus islandicus* rod-shaped virus 2 can be used in bioconjugation chemistry [12,13,14]. Such a useful side of viruses is an object of close research attention, allowing new horizons for their possible applications.

Viruses and noninfectious virus-like particles exhibit the characteristics of ideal building blocks with perfect symmetry and with uniformity of size and shape, which is due to the precise assembly of hundreds of molecules into highly organized scaffolds [15]. Of particular interest is the use of viral particles in the phage display preparation of recombinant antibodies. Phage display is based on the expression of foreign peptides or proteins on the surface of phage particles as part of a chimeric envelope protein [16,17,18]. George P. Smith created this technology in the middle of the 1980s after demonstrating that a foreign protein could be expressed on the surface of bacteriophage M13 (filamentous bacteriophage). He combined the pIII minor coat protein of M13 with the gene encoding the EcoRI restriction endonuclease fragment in a single translation frame [19,20,21,22]. British biochemist Sir Gregory Winter employed phage display to display antigen-binding immunoglobulin fragments on the surface of bacteriophage fd in the 1990s [23]. A fresh combinatorial strategy for preparing recombinant antibodies was thus developed.

The goal of antibody phage display is to create phage particles that show antibodies or their fragments on the membrane with a high degree of specificity to the target antigen or high-affinity phage antibodies. The three main steps of this technology are as follows:

- Making bacteriophages or choosing them from libraries of available phages;
- Biopanning (affinity-based enrichment of phage libraries);
- Phage-displayed antibodies go through a number of affinity selection procedures before being utilized as specific biosensor receptors [24].

Since the beginning of the new millennium, viruses have considerably worsened the global epidemiological situation. Examples include the spread of severe acute respiratory syndrome (SARS) in 2002–2004, the A/H1N1 swine flu pandemic in 2009, and the Ebola outbreak in West Africa in 2014 [25]. In 2019, the world was shaken by the coronavirus pandemic (COVID-19), which has reached unprecedented proportions and has made substantial adjustments to everyone’s habitual way of life [26,27]. According to the World Health Organization (WHO), 763,740,140 cases of COVID-19 infection and 6,908,554 deaths have been confirmed in 230 countries and territories worldwide (as of 20 April 2023) [28]. In addition, complications in the form of unregulated immunological responses, metabolic dysfunction, and multiple organ failure have been reported in patients with acute COVID-19 [29].

On 20 May 2022, WHO released a full report on global health statistics for 2020, the first year of the COVID-19 pandemic that resulted in 4.5 million excess deaths. The statistics show that the pandemic affected the health care system worldwide, in some cases severely limiting access to life-saving resources. The pandemic has significantly slowed the global progress in both life and healthy life expectancy that was achieved in the first 20 years of the century. The report highlights that the global community has been unprepared to recognize the central role of primary health care and to fully fund key elements of health care, which has largely slowed the effectiveness of the response to the spread of the SARS-CoV-2 virus [30].

On 12 August 2022, the United States Center for Disease Control and Prevention reported two new human cases of the H3N2 influenza virus circulating in swine. The outbreaks of influenza A subtype H3N2 among pigs in the United States indicate that the risk of human infection with this virus and its further spread may now be higher than usual. Since the monkeypox outbreak began and as of 3 January, 21,094 confirmed cases have been reported in 29 countries. Five deaths were recorded during the spread of monkeypox: three in Spain, one in Belgium, and one in the Czech Republic [31].

With increased population mobility and international travel, viruses spread rapidly around the world, causing outbreaks of infectious diseases. The emergence of a new virus on a pandemic scale threatens the health and lives of 8 billion people. A major reason for the high prevalence of viral diseases is the lack of effective methods of their detection, including preventive detection in the absence of visible disease symptoms. Therefore, there is a growing need to develop highly sensitive and selective virus detection methods that would be suitable for a wide range of applications, including disease diagnosis, pharmaceutical research, agriculture, and preventive measures.

We describe the advantages of phage display technology in the production of virus-specific antibodies, and we review what is known about virus detection with phage antibodies used in standard and sensor-based methods.

## 2. Methods of Virus Detection

**Figure 1:**
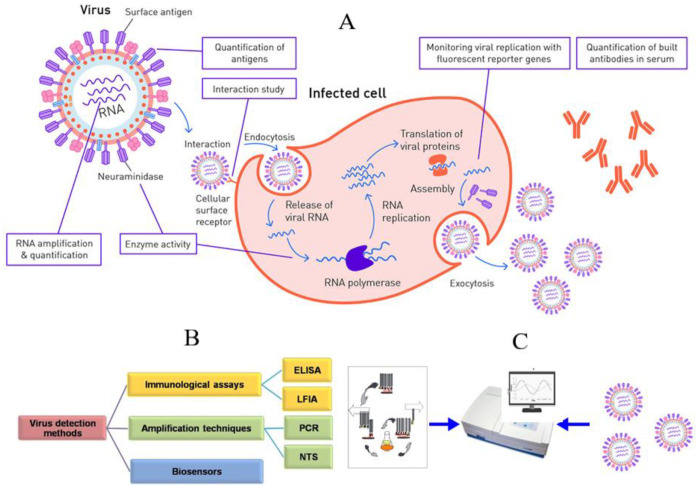
Viral lifecycle in a eukaryotic cell (**A**), virus detection methods (**B**), and the overall scheme of antibody phage display technology for sensor-based virus detection (**C**) [32] with modification.

Many methods are used to identify viruses and virus-like particles. The most common traditional virus detection methods are time-consuming, expensive, often difficult to reproduce, and require special instrumentation and qualified personnel [32]. Virus detection methods facilitate the recognition of different virion components and fractions. Such targets may include fragments of the viral genome, overexpressed antigens, enzymes, and so on (Figure 1).

One “gold standard” method for virus detection is the isolation of pathogens from infected cell cultures [33,34]. Virus isolation can be a very reliable method, but it takes several days to several weeks to produce results and requires trained personnel and expensive equipment. Another “gold standard” method is the polymerase chain reaction (PCR), used in the laboratory diagnosis of viral infections [35,36,37,38,39]. By amplifying the target nucleic acid in the presence of intercalating fluorescent dyes such as SYBR Green or SYTO-13, semi-quantitative detection results may be obtained in a few hours. However, PCR requires a precise thermocycling regimen and, as a rule, special equipment.

Yet another “gold standard” method is the enzyme immunoassay (ELISA), which is often used to detect infections caused by human immunodeficiency virus (HIV), dengue virus (DENV), and other viruses [38,40,41]. The detection by ELISA of viral antigens in clinical samples is limited by the insufficient sensitivity of the assay. Despite these disadvantages, PCR and ELISA enable real-time detection of viruses.

**Figure 2:**
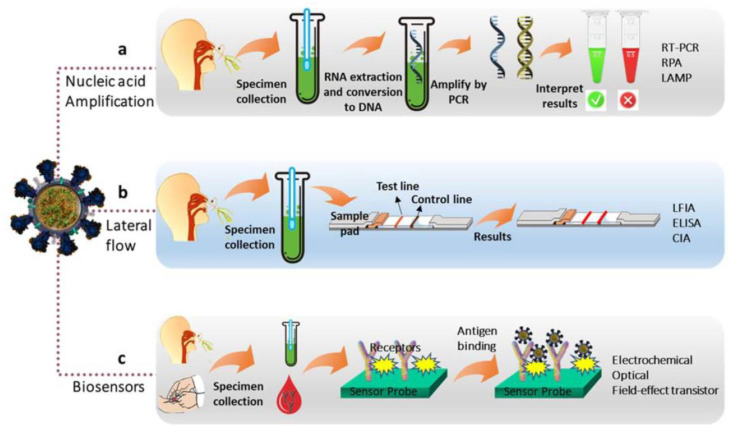
Methods used to diagnose COVID-19. (**a**) Molecular testing based on nucleic acid amplification assays, (**b**) lateral flow immunoassay, and (**c**) biosensors [46].

In recent years, DNA microarray technology has been used to diagnose various viral infections, particularly acute respiratory infections [42,43,44]. In addition, the lateral flow assay is very popular for virus diagnosis [45]. The use of immunochemical and serological methods is also hampered by the high antigenic diversity of some virus groups. Table 1 compares the methods traditionally used to detect pathogenic viruses [26]. Owing to these limitations, traditional methods do not allow simultaneous and sensitive detection of viruses and virus-like particles in clinical samples. Figure 2 shows the main diagnostic methods for COVID-19 [46].

**Figure 3:**
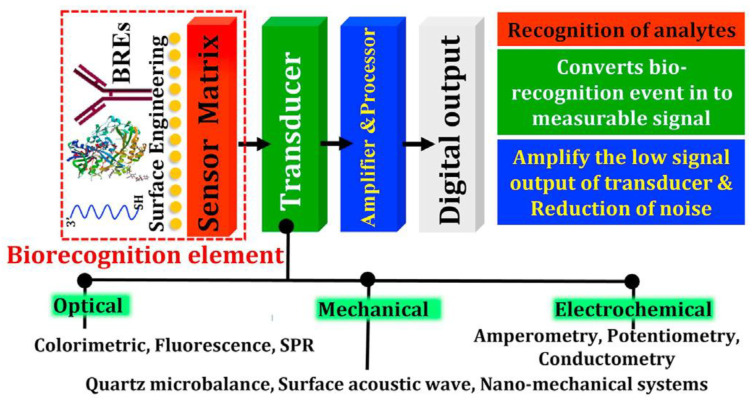
General design scheme of biosensors [47].

One of the most promising tools for virus detection is biosensor-based analysis methods. Biosensors consist of a biologically sensitive element (receptor), a transducer, and a working solution (Figure 3).

The variety of biological materials and methods for their immobilization on the transducer surface has led to the development of three main categories of biosensors, depending on the type of bioreceptor [48]:

- Enzymatic biosensors;
- Affinity biosensors;
- Cell/tissue biosensors.

One immunoanalytical method is affinity biosensors, which track the development of an antigen-antibody combination [48,49]. The efficacy of biosensors depends on such factors as the type of immobilization of recognition elements (biomolecules), with preservation of their natural activity; the availability of the recognition element to the corresponding analyte in solution; and the low nonspecific adsorption on a solid carrier. Therefore, high requirements are imposed on the recognition molecules of biosensors. Specifically, the ligand must be highly specific, chemically and physically stable, and economical to produce. For this reason, antibody-based biosensors as sensitive detection elements are the most common ones.

**Figure 4:**
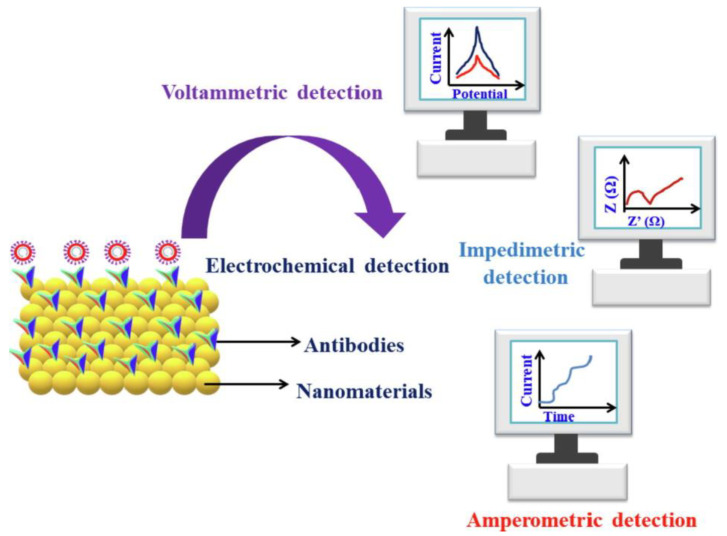
Electrochemical techniques used for SARS-CoV-2 detection [50].

Kumar et al. [50] showed the prospects for the application of electrochemical sensors in virus detection. Amperometric immunosensors, which combine the principles of voltammetry with immunological responses, are highly sensitive in detecting immune interactions. Figure 4 shows the electrochemical techniques used for SARS-CoV-2 detection [50].

Zhang et al. [51] described an electrochemical method for the detection of H5N1 avian influenza virus. The method uses antibodies immobilized on magnetic nanoparticles through a sandwich immunoassay. Through a sandwich immunoassay, antibodies are fixed on magnetic nanoparticles in the procedure. By electrochemically producing protons from water, the magnetic nanoparticles are transformed into an electroactive counterpart of Prussian blue, releasing Fe^3+^. The reduction of deposited K_3_Fe(CN)_6_ and Fe^3+^ to K_4_Fe(CN)_6_ and Fe^2+^, respectively, in a subsequent electrochemical step at a lower potential, results in a reaction that creates a Prussian blue analog). This method detects a much lower analyte concentration than traditional methods.

**Figure 5:**
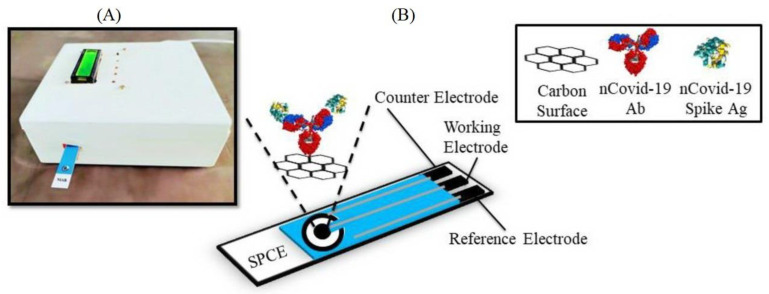
(**A**) Fabricated electrochemical eCovSens device; and (**B**) fabrication of an SPCE electrode, in which nCovid-19 antibody (Ab) is immobilized onto the transducer of the SPCE. The transducer detects changes in the electrical signal resulting from the antigen–antibody (Ag–Ab) interaction [52].

Another antibody-based electrochemical device was described by [52] (Figure 5). The sensor system is based on measuring changes in conductivity by immobilization of a monoclonal antibody to SARS-CoV-2 on screen-printed carbon electrodes. The performance of both electrodes was measured by the interaction of the monoclonal antibody with the specific spike protein of SARS-CoV-2. The limit of detection was 120 fM, whereas it was 90 fM with an in-house built biosensor device (eCovSens) in the case of spiked saliva samples. The advantage of eCovSens is that it can detect the SARS-CoV-2 spike antigen within 10–30 s.

Białobrzeska et al. [53] described an immunosensor for the detection of SARS-CoV-2 N protein sequences on the basis of nucleocapsid antibodies against SARS-CoV-2. The antibodies were prepared and purified in the laboratory and were tightly immobilized on different surfaces (diamond/gold/glass carbon). The biosensor allowed rapid detection of the SARS-CoV-2 virus (detection time, less than 10 min), with detection limits ranging from 0.227 ng/mL (glass carbon) to 0.334 ng/mL (diamond) and 0.362 ng/mL (gold).

**Figure 6:**
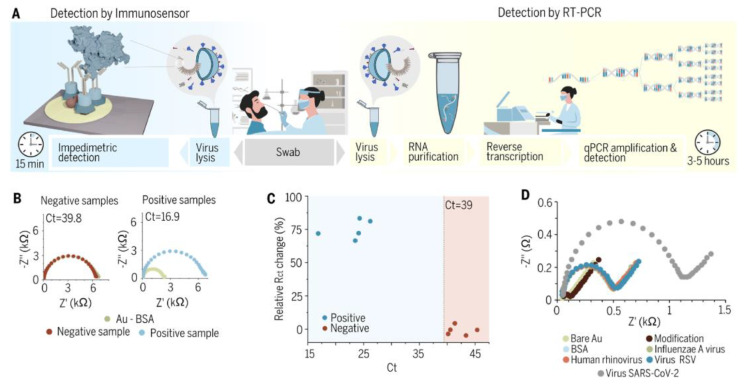
Clinical diagnostics with gold immunoassays. (**A**) Schematic comparison of the conventional qRT−PCR detection methods and the point-of-care ultrafast immunoassay. (**B**) Impedimetric spectra for the gold sensor were acquired when it was incubated with a mixture of positive samples (saliva swabs containing SARS-CoV-2) and negative samples (saliva swabs missing SARS-CoV-2 but carrying other pathogens (three samples) or from healthy individuals (two samples). (**C**) Evaluation of the tested samples’ Ct and ΔRct values. (**D**) Testing the antibody cross-reactivity with various upper respiratory tract viruses, including human rhinovirus, influenza A virus, and respiratory syncytial virus [53].

For all surfaces tested, a wide linear range of analyte concentrations was obtained (4.4 ng/mL–4.4 pg/mL). Figure 6 compares the developed method with the immunoassay, the “gold standard” in virus detection.

Using the detection of bacteriophage T7 as an example, Lesniewski et al. [54] proposed a colorimetric immunosensor based on gold nanoparticles covalently bound to antibodies specific to this bacteriophage. Owing to the formation of immune complexes of phage particles with antibodies and gold nanoparticles, the immunosensor permitted fast, simple, and selective detection of the virus. As a result, one can observe a solution color change from red to purple with the naked eye. The authors conclude that this method can be used to detect almost all viruses and virus-like particles.

While the first electrochemical devices were being developed, optical sensors, including planar waveguide sensors, were also being developed at the same time [55]. Internal and exterior optical fiber sensors can be split into two major categories. Thus, a feature of internal sensors is that the external force that acts on the fiber can change not only the transit time but also the intensity/polarization of the light propagating down the fiber.

**Figure 7:**
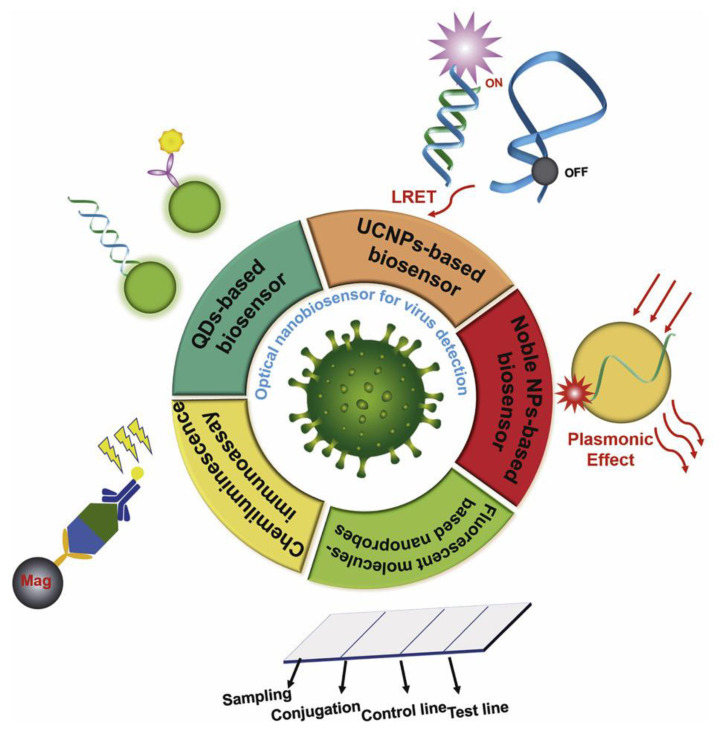
Schematic diagram of optical nanobiosensors used to detect pathogenic viruses. These biosensors are designed on the basis of quantum dots, upconversion nanoparticles, noble metal nanoparticles, 2D nanoprobes based on fluorescent organic molecules, and chemiluminescence immunoassay [26].

The advantages of optical nanobiosensors include (1) high sensitivity along with an ultralow limit of detection; (2) the ability to modify the analysis for a specific sample, including when the signal is visualized directly with the naked eye for the rapid reading of results and subsequent diagnosis; and (3) the relatively low cost of the portable laser and, therefore, the biosensor itself (Figure 7) [26].

An optical immunosensor was developed to detect the NS1 antigen (nonstructural protein 1) as a biomarker of dengue virus in clinical samples obtained early in infection. The principle of operation is based on the detection of the NS1 antigen by immunofluorescence by using fluorescein-5-isothiocyanate (FITC) conjugated to an IgG antibody. The probe was highly reproducible (relative standard deviation, 2%) and sufficiently stable for 21 days at 4 °C, with a detection limit of 15 ng/mL^−1^ [56].

Guliy et al. [57] reported on the optical sensor-aided immunodetection of transmissible gastroenteritis virus (TGEV). The analysis is based on the measurement of changes in the electro-optical variables of the sensor before and after the addition of TGEV-specific antibodies to the suspension being analyzed. The measuring process can be fully automated, and it is possible to detect TGEV in the presence of foreign viral particles.

Owing to their excellent chemical stability, acoustic biosensors are most frequently made with piezoelectric crystals such as quartz, lithium niobate, and lithium tantalate. An attractive approach to creating a family of sensors characterized by high sensitivity, rapidity of analysis, low cost, and tiny sizes is the excitation of acoustic waves in a piezoelectric material. Some techniques rely on membranes or receptor antibodies placed on a piezoelectric waveguide or the resonator surface [58]. For instance, immunosensors were developed for the selective detection of the herpes virus in human blood [59] and in natural water reservoirs (rivers, sewers, wastewaters) without the need for preprocessing of the analyzed substrate [60]. These immunosensors immobilized the appropriate antiviral antibodies on the surface of a piezoelectric resonator.

For example, bacteriophage M13 was identified in real-time by Tamarin et al. [61], who used elastic Love waves with horizontal shear polarization in a layered medium. First, phage-specific antibodies were permanently fixed on a silicon oxide substrate. The multilayered structure that had developed on the waveguide surface was then examined after an immunoreaction between the bacteriophage and the immobilized antibodies was induced. The formation of the structure led to a change in the velocity and attenuation of the Love wave. The particles bound to the sensor surface antibodies (control for counting the phage titer) were eluted, and the pH of the solution was changed. Plaque-forming units (PFU) were counted by microbiological methods.

An electroacoustic sensor platform was used for the immunodetection of the transmissible gastroenteritis virus of swine (TGEV) [62]. Recently, colorimetric sensors [63] and surface-enhanced Raman spectroscopy (SERS) [64] have been actively used for virus diagnosis.

Significantly, all immunochemical methods, including biosensor-based ones, depend primarily on the quality of the specific antibodies used to make diagnostic systems. Antibody selection targets range from peptides and recombinant proteins to viral or virus-like particles [65]. Virus-specific antibodies can be derived from libraries generated from different animal species, such as macaque [66] and chimpanzee [67] monkeys, llamas [68], mice [69], chickens [70], and humans [71].

## 3. Phage Display Technology

Historically, the first source of antibodies was sera from immune animals and humans. The antibodies obtained contain a set of immunoglobulins of different classes and subclasses that specifically recognize their antigen. Different serum antibodies recognize several sites (epitopes) of the antigen. Antibody preparation was then aided by hybridoma technology [72], which makes it possible to obtain antibodies that are produced by a single-cell clone, recognize a single epitope, and retain their properties in many hybrid-cell generations. The advantages of monoclonal antibodies include their high specificity and the possibility to prepare large quantities of antibodies with a given specificity. However, the technology for preparing monoclonal antibodies was developed for mouse and rat studies and is difficult to apply in the case of human antibodies, whereas for therapeutic purposes, human immunoglobulins are required. Difficulties also arise when immunization of animals, for whatever reason, is impossible or the lack of immunogenicity of potential antigens cannot be overcome. Finally, monoclonal antibodies cannot be used with traditional and popular immunological methods based on the precipitation reaction.

To resolve these problems, methods for the molecular cloning of antibody gene fragments have been put forward. One such method is antibody phage display, proposed by [23]. The antigen-binding fragments (scFv, Fab) presented on the surface of a filamentous bacteriophage can be selected on an immobilized antigen. The main idea behind the method is to design a combinatorial library in which the variable regions of the light and heavy chains of immunoglobulins are combined randomly and are presented on the surface of the bacteriophage. Every bacteriophage, such as a B lymphocyte, expresses antibodies of certain specificity. If the size of a library is large enough, the repertoire of variable regions will be comparable to that of antibodies in the body. The advantages of the method are as follows:

- Antibodies can be selected in vitro, and animal immunization can be omitted;
- No need to use laboratory animals or maintain long-term cultures of eukaryotic cells;
- The preparation of individual clone producers of miniantibodies takes 10–14 days, against the several months that hybridoma technology takes;
- Antibodies are relatively easy to prepare, and their cost is low;
- It is possible to make hybrid molecules with marker proteins (e.g., tag peptide) in a short time;
- It is possible to prepare antibodies to autoantigens, weakly immunogenic compounds, and toxins;
- The method can be used in immunotherapy.

Temperate filamentous phages (M13, f1, fd, etc.) are most commonly used in phage displays. Their virion contains circular single-chain DNA, whose genetic organization and sequence are known by many members of this phage group. The genome size is about 6000 nucleotides. The five structural proteins that make up the capsid of a viral particle are organized similarly in all members of the group [23].

Human phage display libraries or libraries from other species are used to make antibodies. These libraries will be utilized for the in vitro selection (biopanning) of the target molecule. The following actions are part of the process of choosing phage library components:

- The immobilized antigen is incubated with library clones;
- Particles of the phage that have not yet attached to the antigen are washed away;
- The bound phage particles are eluted;
- The selected clones are infected with bacterial cells (*E. coli*);
- The affinity clones are amplified and isolated.

**Figure 8:**
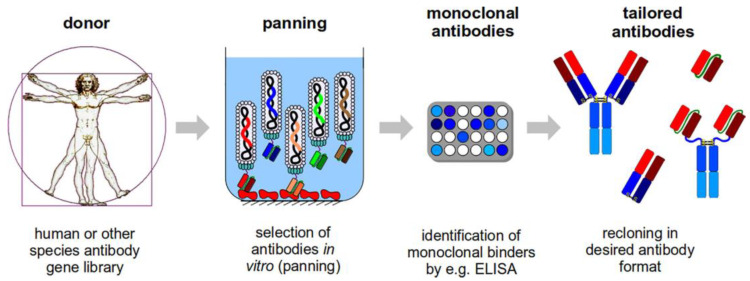
Scheme for antibody preparation [65].

The immunogenicity of the antigens, how they are immobilized, and the quantity of biopanning rounds all affect how long it takes to create an antibody. That is why the process can take a few hours or several days. After biopanning, the monoclonal phage antibodies are analyzed by various immunochemical assays, in particular, ELISA. Subsequently, the genes of the selected antibody fragments can be isolated from the phage particles for subsequent cloning and expression. Figure 8 illustrates the antibody generation process [65]. The availability of the antibody library for screening is the primary prerequisite for antibody phage display. Antibody libraries, depending on how they are constructed, fall into four main categories—naïve, immune, semisynthetic, and synthetic.

The following is the general process for creating a combinatorial phage library:

- The genes of scFv, Fab, or other fragments are cloned from immune or undamaged humans, mice, rabbits, chickens, pigs, dogs, monkeys, sheep, or cows’ B lymphocyte mRNA;
- These genes, along with the gene coding for the capsid protein (typically p3), are introduced into the phagemid in a single translation frame;
- Phagemid genes are expressed, and virions are put together in infected *E. coli* cells by using the generated phagemid repertoire.

Foreign antibody fragments will be exposed as part of the capsid proteins of the virions. Depending on the chosen vector system, this step takes place with or without the use of helper phages.

In this way, a population of bacteriophages is prepared, each exhibiting a specific antigen-binding domain on its surface [73,74]. The application of phage antibodies in bioreceptors is particularly promising. Phage antibodies themselves are faster and less labor-intensive to prepare than those prepared by hybridoma technology. Phage antibodies have a number of benefits over their natural counterparts, including the following:

- Owing to the Fc region of the intact antibody, the tiny size of the antibody fragments is typically accompanied by reduced nonspecific binding;
- The biosensor can immobilize phage antibodies more densely;
- Phage antibodies, as opposed to full-length antibodies, are produced by *E. coli* cells, which significantly lowers production costs because no specialized equipment is needed for the culture of hybridoma cells [75].

## 4. Phage Antibodies for Virus Detection/Identification

Phage display technology antibodies as nano-bio-engineered macromolecules can be used for sensor-based virus detection. In order to recognize lethal viruses such as Zika, Ebola, Hendra, Nipah, Hanta, Middle East respiratory disease (MERS), and SARS, phage display has been utilized to create new diagnostic tools [76,77]. In the current situation of the COVID-19 pandemic, researchers actively search for neutralizing antibodies against SARS-CoV-2 for therapeutic use. Because phage display is an important antibody selection method, the prospects for the use of phage display, with special emphasis on its use in the diagnosis and therapy of coronavirus diseases, were shown by Anand et al. [77].

By now, a huge pool of antibodies against various viruses has been obtained by phage display, by using naïve or immune libraries. From naïve antibody gene libraries, antibodies against human pathogenic viruses such as SARS coronavirus, dengue virus, influenza virus, Venezuelan equine encephalitis virus, norovirus, and hepatitis C virus have been developed by using recombinant viral proteins or complete viral particles [78,79]. Other antibodies have been chosen from immune antibody gene libraries that target the influenza virus, HIV, SARS coronavirus, yellow fever virus, and Western equine encephalitis virus [79]. Even semisynthetic libraries have been utilized to prepare antibodies specific to the influenza virus [80]. In the past, libraries from several species have been used to successfully isolate virus-specific antibodies.

By using phage display, potential therapeutic agents are being developed for other human coronaviruses, such as SARS [81] and MERS [82], and for animal coronaviruses, such as infectious bronchitis virus, TGEV, and porcine epidemic diarrhea virus. High-affinity/small-molecule antibodies have been identified for the E, N, and S proteins of SARS-CoV [77]. For example, Ubah and Palliyil [83] described the advantages of phage antibodies and the prospects for their use against various viral targets. Other VHH segments produced via phage display and animal immunization include those that bind to *Rotavirus* gp6, H5 hemagglutinin to inhibit H5N1 influenza virus replication, and VHH that recognizes the tail of infectious phage in *Lactococcus bacteria*.

Examples of the use of phage antibodies for virus detection using classical methods are presented in Table 2.

Undoubtedly, phage antibodies are an excellent alternative to classical antibodies as sensing elements in virus diagnostics by biosensors (Table 3). For example, Kim et al. [180] developed a COVID-19 biosensor based on the lateral flow immunoassay (LFIA). They used three rounds of biopanning to generate phage antibodies (from a naïve chicken phage library) with a single-chain variable fragment (scFv) and crystallizable fragment (Fc) combination, which was specific for SARS-CoV-2 nucleocapsid protein. The scFv–Fc antibodies bound specifically and with high affinity to the nucleocapsid protein of SARS-CoV-2 but not to those other coronaviruses. The detection limit for this virus was 2 ng of antigenic protein. The biosensor could selectively detect SARS-CoV-2 virus within 20 min without detecting SARS-CoV or MERS-CoV virus. In addition, the assay was made more accurate with a portable LFIA reader. When the LFIA biosensor was mounted onto a portable reader, the obtained image was analyzed automatically with LED and CMOS sensors [180].

A sandwiched phage-based enzyme-linked chemiluminescence immunoassay was created by Liu et al. [195] by using the discovered phage expressing a particular peptide as a bifunctional probe. The probe was capable of recognizing the SARS-CoV-2 S1 antigen and amplifying the signal. The method is useful for the detection of SARS-CoV-2 pseudovirus in saliva.

After three rounds of biopanning, Yang et al.’s [199] screening of a phage library for affinity to the SARS-CoV-2 spike protein produced five SARS-CoV-2-specific phage miniantibodies. The resulting antibodies were used to develop a rapid and cost-effective test for the detection of the SARS-CoV-2 virus. With the aid of a handy fluorometer, phage antibodies were successfully transformed into fluorescent immunosensors that allowed quick detection of the SARS-CoV-2 viral antigen [196]. From a combinatorial library, high-affinity synthetic antibodies were isolated by phage display and tagged with gold nanoparticles [197]. These antibodies were successful in identifying the SARS-CoV-2 spike-protein receptor-binding domain and the glycoprotein released by the Ebola virus.

A human combinatorial scFv antibody library was used to retrieve a panel of recombinant single-chain antibodies (scFvs) against structural proteins of tomato spotted wilt virus via phage display [200]. Using phage display to create recombinant antibodies, Dong et al. [201] investigated the specificity of clone binding to the CS protein and hemagglutinin of the H5N1 avian influenza virus. It has been shown that the antibodies identified only epitopes distinct from those seen in other influenza subtypes, enabling quick identification of the H5N1 virus. In order to quickly and accurately detect the H5N1 virus, these recombinant Fab fragments can be employed as a receptor component of sensor systems.

To distinguish avian influenza virus (H5N1) from seasonal influenza viruses (H1N1 and H3N2) (whose nucleoproteins are 90–94% similar in amino acid sequence), Yu et al. [202] developed a phage display-based methodology for obtaining antibodies as affinity reagents against closely related influenza virus nucleoprotein subtypes.

The HBsAg of the hepatitis B virus was demonstrated by ELISA to have a high affinity for the generated phage scFvs, which allowed them to bind to the antigen on the membrane of the infected cells. One of the clones was capable of being internalized into HepG2.2.15 cells that were HBsAg positive, according to indirect fluorescent labeling examination. The ability to internalize phage antibodies to deliver medications to cells that are infected with the hepatitis B virus may be highly promising [120].

By using phage display, methods have been developed to generate specific single-chain variable fragments (scFvs) against dengue fever virus [203] and infectious bursal disease virus (IBDV) [204]. The prepared miniantibodies were used in ELISA to detect viruses in the sera of infected people.

Phage antibody-based ELISA versions have been created for the detection of human disease-causing viruses such as HIV, herpes simplex virus, poliovirus, polyomavirus, rabies virus, respiratory syncytial virus, rotavirus, Sin Nombre virus, Usutu virus, West Nile virus, yellow fever virus, Zika virus, human metapneumovirus, Japanese encephalitis virus, MERS, norovirus, paramyxovirus, chikungunya virus, Ebola virus, enterovirus 71, Epstein-Barr virus, hantavirus, human cytomegalovirus, Hendra virus, Nipah virus, and hepatitis A, C, and E viruses [65].

A useful natural resource for investigating polyvalent interactions with particular antibodies is bacterial viruses. Such research is very useful in the real world and has been used to create improved virus detection methods. For example, Guliy et al. [198] showed the possibility of bacteriophage FAI-Sp59b detection in the presence of foreign viral particles.

The possibility of using phage display antibodies in veterinary medicine was demonstrated in [74]. The great selection potential of phage display antibodies allowed mouse scFvs to recognize the avian influenza virus with excellent sensitivity and specificity [140]. It should be noted that the IBDV virus seriously harms the chicken industry. Owing to the diversity of IBDV strains, it has long been challenging to distinguish between the very virulent classical variation of IBDV (vvIBDV) and the vaccine strain. A single-chain variable fragment that effectively identified the highly conformational epitope of the VP2 protein of vvIBDV was obtained by Sapats et al. [205]. These phage antibodies are perfect for diagnostic techniques such as ELISA since they successfully distinguish vvIBDV from other strains [205,206]. Phage antibodies and an immunoassay were used in related research to find duck hepatitis A virus (DHAV) [99].

One of the most destructive viral illnesses in the dairy and meat sectors is foot-and-mouth disease (FMD). It is known that there are seven FMD serotypes: O, A, C, Asia1, SAT1, SAT2, and SAT3. Therefore, a quick and precise way of differentiating between vaccinated and infected animals is essential to the effectiveness of diagnosis and vaccination. Recombinant chicken scFvs against the FMD 3ABC protein have been produced and demonstrated to be effective for separating infected from vaccine-exposed animals via phage display [110,207]. Additionally, mouse scFvs directed against the VP2 protein of the FMD virus showed a diagnostic capability for a number of serotypes of this virus [113]. For the serological detection of bovine immunodeficiency virus in cattle, recombinant mouse scFv antibodies were utilized in an ELISA with competitive inhibition [87].

The promise of phage antibodies as an experimental tool has been demonstrated to visualize [159] and diagnose [161,208] porcine epidemic diarrhea virus, to diagnose canine parvovirus type 2 (CPV2) [91], and to detect Western and Venezuelan equine encephalitis viruses [189,209].

Possibilities have been shown to detect plant viruses (broad bean mottle virus, grapevine leafroll–associated virus 3, cucumber mosaic cucumovirus, plum pox virus, and grapevine virus B) and zoonotic viruses (Australian bat lyssavirus, bovine viral diarrhea virus, bluetongue virus, classical swine fever virus, canine parvovirus, hematopoietic necrosis virus, ectromelia virus, porcine circovirus, Newcastle disease virus, simian immunodeficiency virus, porcine reproductive and respiratory syndrome virus, transmissible gastroenteritis virus, swine influenza virus, white spot syndrome virus, and vaccinia virus) with phage antibodies and ELISA [65,74].

Norovirus, Maedi-visna, SARS-CoV, Sin Nombre, and duck hepatitis A viruses have been identified with the help of dot-blot immunoassay and phage antibodies [65,74]. Phage antibodies are employed in immunochromatographic assay techniques to diagnose infections brought on by the avian influenza virus, SARS-CoV-2, and dengue virus [65,74].

Note that surface plasmon resonance (SPR) sensors for the detection of chemical and biological species are often the “gold standard” [210,211,212]. SPR biosensors are also used for virus detection. Phage antibodies were used as part of the SPR biosensor for the detection of norovirus [154], porcine circovirus [159], cowpea mosaic virus [213], SARS-CoV-2 [214], etc.

As can be observed from the data displayed, biosensors enable a large decrease in the analysis time owing to the relative simplicity of the procedures, are quite sensitive, and require little pretreatment of the material under examination. The instrumental implementation of these methods should ensure high accuracy of measurements, and in turn, measurements should be made automatically by moderately skilled personnel. When biosensors are designed for the detection of viruses and virus-like particles, the following criteria should be taken into account: property of being all-purpose, ability to be used with various objects, rapidity of detection, specificity, sensitivity, portability, simplicity, ability to use small sample volumes, and low cost [215].

## 5. Discussion and Prospects

The selection of antibody fragments from phage display libraries thus offers the potential to access very large numbers of molecules with different binding specificities quickly and cheaply, avoiding the need for animal immunizations. The scFv can be genetically engineered to produce tailored constructs as described above. Such phage antibodies can be coated directly onto metal, plastic, silica or carbon surfaces by simple adsorption, which, as discussed above, is adequate for biosensor-based assays.

Viruses are pervasive life forms that need a host to reproduce. From bacteria to plants and animals to viruses, a wide variety of organisms may become infected. The genome, which is represented by double- or single-stranded DNA or RNA, and the capsid are the two primary structural parts of viruses. A small number of proteins encoded by the viral genome are present in numerous copies to create the extremely symmetrical capsid [216]. Methods for virus detection are employed in contemporary virological research for a variety of applications. They can be used in epidemiology to track and manage pandemic outbreaks of illnesses caused by viruses, including swine flu, SARS-CoV-2, and many others. Diagnostic virus detection methods are central in controlling the spread of viruses and help contain viral infections. Despite its drawbacks, the use of PCR to identify particular viral genomic sequences while an infection is still active is still regarded as the best virus detection technique. However, sensor technologies using phage antibodies as sensitive elements are very promising because antibodies generated by phage display are an excellent alternative to classical antibodies. Since its development in 1985, phage display has been a crucial and successful molecular biology technique that has remained essential for the scientific community. A phage library may include millions, or perhaps billions, of distinct and distinctive mapping peptide ligands because a huge number of nucleotide fragments can be cloned into the phage genome. The epitope mapping and antigen presentation on the bacteriophage surface, which constitute the basis for phage display, have been exploited in affinity selection-based biopanning to screen for potential novel vaccine candidates [77].

Phage display is a powerful tool for target ligand selection owing to its being simple, highly effective, rapid, and cheap. Efficient biopanning selection leads to the isolation of ligands with unique, specific, and desired functional characteristics. Phage antibody display offers multiple platforms for the use of antigen-binding; however, the diversity and stability of the library still need to be improved.

In the past few years, the need for highly sensitive bioanalytical methods to diagnose and monitor socially significant diseases has increased. This is due, on the one hand, to the growth of the disease in general, including the unprecedented spread of the new coronavirus infection, SARS-CoV-2, and, on the other hand, to the need to overcome the limitations of current biomedical diagnostic methods. For example, because of puncture invasiveness, a tissue biopsy cannot always be performed, and the results of a single biopsy often cannot provide sufficient information in real-time to diagnose the disease. PCR is currently one of the most popular tools for the rapid detection of viral infections. Nucleic acid-based virus detection usually provides high sensitivity but may require trained personnel and be time-consuming and expensive. The use of isothermal amplification systems may reduce equipment costs, making these systems indispensable when high-performance and rapid cycling testing are required. Alternatives to PCR are immunoassays, which offer reliability and cost-effectiveness. In addition, some immunoassays can be modified with lateral flow technology, which greatly speeds up the generation of results. However, immunoassays are usually inferior in sensitivity to PCR. Along with these methods, the use of next-generation sequencing can provide promising results. In addition, the ability to sequence a large number of viral genomes will provide researchers with expanded information about them and will help in tracing infections.

Characteristics such as high throughput, ease of use, and short running time play an important part, especially in viral outbreaks, in addition to the general requirement for the accuracy, validity, and specificity of viral assays. This strategy enables simple, cheap, rapid, and sensitive detection of specific pathogens, which shows great potential in virus analysis in situ.

The growing advances in phage display and the use of antibodies in the diagnosis of viral diseases suggest this technology will be improved further. This progress will gradually drive innovation to understand the mechanisms involved in infection and to design post-exposure therapies for SARS-CoV-2 and other viral infections.

## Figures and Tables

**Table 1:** Comparison of methods traditionally used to detect pathogenic viruses [26].

Technique	Principle	Time	Advantages	Disadvantage
PCR	nucleic acid	hours	- well established - small number of samples	- ease to contamination - time-consuming
ELISA	viral protein	hours	highly specific	- low sensitivity - high-quality sample preparation
Cell culturing	infectivity assay	days to weeks	- suitable for virus sub-typing recovery of novel and divergent strains - inexpensive	- contamination problems - time-consuming and labor intensive - unavailable for immediate patient care
Electron microscopy	viral particles	hours	rapid method	- well-trained personnel - low specificity
Computed tomography	chest scanning	hours	good basis for clinical diagnosis and treatment	- technical expertise - centralized facilities

**Table 2:** Examples of using recombinant antibodies for virus detection.

Virus		Phage Ab Target	Type of Phage Library	Format of Phage Abs	Application of Phage Abs	Reference
Australian bat lyssavirus (ABLV)		glycoprotein G	naïve	Fab, IgG	ELISA, in vitro neutralization	[84]
Avian influenza virus H7N2 (AIV)		complete virus	immune	VHH	ELISA	[85]
Bluetongue virus (BTV)		complete virus	semi-synthetic	scFv, scFv-Fc	ELISA	[86]
Bovine immunodeficiency virus (BIV)		capsid (CA) protein	semi-synthetic	scFv	ELISA, WB	[87]
Bovine viral diarrhea virus (BVDV)		envelope 2 (E2) protein	immune	VHH	ELISA, qRT-PCR, in vitro neutralization	[88]
		nonstructural protein 5 (NS5B)	immune	VHH	ELISA	[89]
Broad bean mottle virus (BBMV)		complete virus	immune	VHH	ELISA, in vitro neutralization	[90]
Canine parvovirus type 2 (CPV2)		complete virus	semi-synthetic	scFv	ELISA	[91]
		virus-like particles (VLP)	immune	scFv	ELISA, ICA, virus suppression assay	[92]
Chikungunya virus (CHIKV)		VLP	immune	VHH	ELISA, MagPlex, in vitro neutralization	[93]
Cucumber mosaic virus (CMV)		complete virus	semi-synthetic	scFv	ELISA, WB	[94]
Dengue virus (DENV)		non-structural protein 3 (NS3)	naïve	Fab	ELISA, in vitro neutralization	[95]
		nonstructural protein 1 (NS1)	immune	VHH	MagPlex assay	[96]
		RNA-dependent RNA polymerase (RdRp) in the viral non-structural protein 5 (NS5)	naïve	scFv	ELISA, WB, in vitro inhibition	[97]
Duck hepatitis A virus (DHAV)		VP3 protein	immune	scFv	ELISA, in vivo neutralization	[98]
		VP1 protein	immune	VHH	ELISA, IF, dot-blot	[99]
Ebola virus		nucleoprotein	synthetic	scFv, IgG	ELISA, WB	[100]
		viable Zaire ebolavirus, viral matrix protein VP40, viral nucleoprotein (NP)	immune	scFv, IgNAR V	ELISA, WB	[101]
		multifunctional viral protein (VP35)	semi-synthetic	scFv	ELISA, WB	[102]
		glycoprotein (GP)	immune	scFv, scFv-Fc	ELISA, in vitro neutralization, in vivo protection	[103,104]
Ectromelia virus (ECTV)		epitope p35	immune	scFv	ELISA, in vitro neutralization, WB	[105]
Enterovirus 71 (EV71)		inactivated EV71 virions	immune	Fab	ELISA, WB, in vitro neutralization	[106]
		virion protein 2 (VP2)	naïve	scFv	ELISA, WB	[107]
		internal capsid protein (VP4)	naïve	scFv	ELISA, WB, IF, in vitro neutralization	[108]
Epstein–Barr virus (EBV)		latent membrane protein 1 (LMP1)	naïve	Fab	ELISA, WB, IF, FACS, in vitro inhibition	[109]
Foot-and-mouth disease virus (FMDV)		recombinant non-structural protein (NSP) 3ABC	immune	scFv	ELISA, WB	[110]
		VLP	immune	VHH	ELISA, IF	[111]
		intact (146S) FMDV	immune	VHH	ELISA	[112]
		VP2 capsid protein	immune	scFv	ELISA	[113]
Grapevine leafroll-associated virus 3 (GLRaV-3)		coat protein	immune	scFv	ELISA	[114]
Grapevine virus B (GVB)		virus particles	semi-synthetic	scFv	ELISA	[115]
Hantaviruses		virus particles	immune	Fab	ELISA, IF, WB	[116]
		nucleoprotein	immune	VHH	ELISA, WB	[117]
Hendra virus (NiV) and Nipah virus (HeV)		attachment envelope glycoprotein G	naïve	Fab, IgG	ELISA, immunoprecipitation, WB, in vitro neutralization, IF, in vivo neutralization	[118]
Hepatitis A (HAV)		HAV capsid	immune	Fab, IgG	ELISA, in vitro neutralization	[119]
Hepatitis B virus (HBV)		hepatitis B virus surface antigen (HBsAg)	immune	Fab, scFv	ELISA, IF	[120]
Hepatitis C virus (HCV)		core protein	immune	Fab	ELISA	[121]
		E2 glycoprotein	immune	Fab	ELISA, in vitro neutralization	[122]
		non-structural protein-3/4A (NS3/4A)	naïve	scFv	ELISA, IF, in vitro neutralization	[123]
		non-structural protein-5A (NS5A)	naïve	scFv	ELISA, WB, IF, in vitro neutralization	[124]
Hepatitis E virus (HEV)		ORF2 protein	immune	Fab	ELISA, WB, in vitro neutralization	[125]
Herpes simplex virus (HSV-1, HSV-2)		glycoproteins gD and gB	immune	Fab	ELISA, immuno precipitation, WB, in vitro neutralization	[126]
Human cytomegalovirus (HCMV)		gycoprotein B (gB) and H (gH)	immune	scFv	ELISA, in vitro neutralization	[127]
		glycoprotein 55 (gp55)	immune	scFv	ELISA, in vitro neutralization	[128]
Human immunodeficiency viruses (HIV)		transmembrane glycoprotein gp41	synthetic	Fab	WB, in vitro neutralization	[129]
		integrase (IN) protein	immune	scFv	ELISA, WB, IF, in vitro neutralization	[130]
		p24	immune	scFv	ELISA	[131]
		envelope glycoprotein gp140	immune	VHH	ELISA, in vitro neutralization	[132]
		CD4bs region of subtype C	immune	scFv	ELISA, in vitro neutralization	[133]
		Envelope glycoprotein gp120	immune	Fab	ELISA, in vitro neutralization, IF	[134]
Human metapneumovirus (HMPV)		F protein	immune	Fab	ELISA, IF, in vitro neutralization, in vivo protection	[135]
Influenza A	H1N1	hemagglutinin protein (HA)	immune	scFv	ELISA	[136]
	H2N2	hemagglutinin protein HA (stem region)	immune	Fab	ELISA, in vitro neutralization	[137]
	H3N2	HA protein and its variants	semi-synthetic	scFv	ELISA	[138]
		non-structural protein-1 (NS1)	naïve	scFv	ELISA, WB, in vitro neutralization, IF	[139]
		nucleoprotein (NP)	immune	scFv	ELISA, WB, in vitro inhibition	[140]
		hemagglutinin protein (HA)	semi-synthetic	scFv	ELISA	[141]
		complete inactivated virus	immune	VHH	ELISA	[142]
Influenza A		M2 protein (cytoplasmatic domain)	naïve	scFv, scFv-Fc	WB, IHC	[143]
Influenza B		whole virus	immune	Fab	ELISA, WB, IF, in vitro neutralization	[144]
		hemagglutinin protein (HA)	immune	VHH	ELISA	[145]
Japanese encephalitis virus (JEV)		purified virion	immune	Fab	ELISA, immunoprecipitation, in vitro neutralization	[146]
		domains I, II, III of envelope protein	immune	Fab, IgG	ELISA, WB, immunoprecipitation, in vitro neutralization, in vivo protection	[67]
Marburg virus		nucleoprotein	immune	sdAbs	ELISA	[147]
		transmembrane glycoprotein (GP)	immune	scFv, scFv-Fc	WB, in vitro neutralization, in vivo protection	[148]
		VP35 protein	synthetic	Fab	ELISA	[149]
Middle East respiratory syndrome-related coronavirus (MERS-CoV)		S2 subunit of the MERS-CoV spike protein (MERS-S2P)	synthetic	Fab, IgG	ELISA, IF, in vitro neutralization, ACCEL ELISA™	[150]
		nucleoprotein (NP)	naïve	scFv	ELISA	[151]
Norovirus		VLP	semi-synthetic	scFv	ELISA, WB	[152]
		VLP with major capsid protein VP1 and a minor structural protein VP2	immune	VHH	ELISA, WB, in vitro inhibition, IF	[153]
		P-domain of the GI.1 (VLP) major capsid protein	semi-synthetic	scFv	ELISA, dot-blot, surface plasmon resonance (SPR)	[154]
Paramyxovirus		glycoproteins F (fusion protein) and HN (attachment protein)	synthetic	Fab, sAb	ELISA, in vitro neutralization, immunoprecipitation	[155]
Plum pox virus (PPV)		NIa protease	semi-synthetic	scFv	WB, dot-blot	[156]
Poliovirus		capsid proteins VP1 and VP3	immune	Fab, IgG	ELISA, in vitro neutralization, in vivo protection	[157]
Polyomavirus		major capsid viral protein 1 (VP1)	synthetic	Fab, IgG	ELISA	[158]
Porcine circovirus type-2 (PCV2)		complete virus	immune	sdAbs	Western blot, ELISA, and SPR	[159]
		cap protein	immune	VHH	ELISA	[160]
Porcine epidemic diarrhea virus (PEDV)		membrane protein of PEDV	immune	sdAb fragments (sdAb-Mc19/29/30/37)	ELISA	[161]
		S1 domain of spike protein	immune	VHH	ELISA, in vitro neutralization	[162]
		nucleocapsid (N) protein	immune	VHH	ELISA	[163]
		S1 region of the spike protein	immune	scFv	ELISA, IF, in vivo protection	[164]
Porcine reproductive and respiratory syndrome virus (PRRSV)		non-structural protein 9 (Nsp9)	immune	VHH	ELISA, IF, immunoprecipitation	[165]
		non-structural protein 4 (Nsp4)	immune	VHH	ELISA, immunoprecipitation	[166]
Rabies lyssavirus		glycoprotein (antigenic site II)	immune	Fab, IgG	ELISA, IF, in vitro neutralization	[167]
		glycoprotein G	naïve	VHH	ELISA, in vitro neutralization, in vivo protection	[168]
Respiratory syncytial virus (RSV)		glycoprotein F	synthetic	Fab, IgG	ELISA, in vitro neutralization	[169]
Rotavirus		non-structural protein Nsp4	semi-synthetic	scFv	ELISA, WB	[170]
		VP8* fraction of rotaviral VP4 outer capsid	semi-synthetic	scFv	ELISA, WB, in vitro inhibition	[171]
Severe acute respiratory syndrome coronavirus 2 (SARS-CoV-2)		S protein (RBD)	synthetic	VH	ELISA, in vitro neutralization	[172]
			immune	scFv, scFv-Fc, IgG	ELISA, in vitro inhibition, in vitro neutralization, in vivo protection	[173]
			naïve	scFv	ELISA, in vitro neutralization, in vitro inhibition, in vivo protection	[174]
			synthetic	Fab, biospecific Fab+VH	in vitro neutralization	[175]
			immune	VHH, VHH-Fc	ELISA, in vitro neutralization	[176]
		RBD	immune	VHH	ELISA, in vitro inhibition, in vitro neutralization	[177]
			semi-synthetic	Fab	ELISA, in vitro neutralization	[178]
			immune	Fab, IgG	ELISA, in vitro neutralization assay, in vivo protection	[179]
		nucleocapsid protein (NP)	immune	scFv, scFv-Fc	ELISA, WB, dot-blot, lateral flow strip assay	[180]
Sin Nombre orthohantavirus (SNV)		Sin Nombre Virus nucleocapsid protein (SNV-N)	naïve	scFv	ELISA, WB, dot-blot	[78]
Simian immunodeficiency virus (SIV)		trimeric (gp140) and monomeric (gp120) forms of the SIVmac239 envelope glycoprotein	immune	scFv, scFv-Fc	ELISA, WB, in vitro neutralization	[181]
Swine influenza virus (SIV)		SIV nucleoprotein (SIV-NP)	immune	VHH	ELISA, WB	[182]
Transmissible gastroenteritis virus (TGEV)		whole virus	immune	scFv	ELISA, in vitro neutralization, IF, WB	[183]
Usutu virus (USUV)		domain III (DIII) of the USUV E protein	immune	scFv	ELISA, WB, in vitro neutralization	[184]
Vaccinia virus (VACV)		virus particles	immune	scFv, IgG	inhibition ELISA, in vitro neutralization	[185]
Venezuelan equine encephalitis viruses (VEEV)		viral E1 envelope protein	immune	scFv, scFv-Fc	ELISA, WB, in vitro neutralization, in vivo protection	[66]
White spot syndrome virus (WSSV)		virus particles	immune	scFv	ELISA, in vitro neutralization	[186]
West Nile virus (WNV)		domain IIII of WNV envelope (E) protein	immune	Fab	ELISA, IF, in vitro neutralization, in vivo protection	[187]
		envelope (E)protein	naïve	scFv, scFv-Fc	ELISA, WB, in vitro neutralization	[188]
Western equine encephalitis viruses (WEEV)		virus	immune	scFv	Enzyme-linked immunosorbent assays (ELISAs)	[189]
		virus particles	immune	scFv, scFv-Fc	in vitro neutralization, in vivo protection	[190]
		E2/E3E2 envelope proteins	immune	VHH	ELISA (MagPlex assay)	[191]
Yellow fever virus (YFV)		domain II of envelope protein	immune	scFv, IgG	in vitro neutralization, in vivo protection	[192]
Zika virus (ZIKV)		envelope (E) protein	immune	scFv	ELISA, WB, FACS, in vitro inhibition	[193]
		nonstructural protein 1 (NS1)	immune	VHH	ELISA	[194]

**Table 3:** Examples of biosensor systems using recombinant antibodies as a receptor element for detecting viruses.

Type of Sensory System	Phage Antibody Format	Virus	Target	Detection Limit	Analysis Time	Reference
Biosensor based on the lateral flow immunoassay (LFIA)	scFv–Fc	SARS-CoV-2	nucleocapsid protein (NP)	2 ng of antigenic protein 2.5 × 10^4^ pfu cultured virus	20 min	[180]
Sandwiched phage-based enzyme-linked chemiluminescence immunoassay (ELCLIA)	n.i.	SARS-CoV-2	S1 domain of the spike glycoprotein	78 pg/mL of antigenic protein	n.i.	[195]
Quench-based fluorescent immunosensor (Quenchbodies)	Fab, Ig	SARS-CoV-2	S1 domain of the spike glycoprotein	0.1 nM of S protein trimer 10^5^ copies/mL of virus	2 min	[196]
Nanobody-functionalized nanoparticles for rapid, electronic detection (Nano2RED)	n.i.	SARS-CoV-2	spike protein receptor binding domain (RBD)	40 pg/mL 1.3 pM	5–20 min	[197]
		Ebola virus	secreted glycoprotein (sGP)	10 pg/mL 0.13 pM		
Electro-acoustic sensor	Ig	bacteriophage FAl-SR65	whole virus particles	10^6^ phages/mL	5 min	[198]

## Data Availability

Not applicable.
